# Efficient HPLC-ELSD analysis of sodium and phosphate in aripiprazole extended-release injectable suspensions using trimodal column technology

**DOI:** 10.1039/d5ra07182h

**Published:** 2025-10-28

**Authors:** Alexandra Bitziou, Theodora Fotiou, Constantinos K. Zacharis

**Affiliations:** a Laboratory of Pharmaceutical Analysis, Department of Pharmacy, Aristotle University of Thessaloniki GR-54124 Greece czacharis@pharm.auth.gr; b Innovative Formulations, Pharmathen S.A. Pharmaceutical Industry Spartis 31 Str., Metamorfosi Attica 14452 Greece

## Abstract

The quantification of inorganic ions such as sodium and phosphate ions in pharmaceutical formulations is crucial for ensuring the consistency and safety of the drug product. This study presents a robust and selective HPLC method coupled with an Evaporative Light Scattering Detector (ELSD) for the simultaneous determination of sodium and phosphate ions in aripiprazole extended release injectable suspensions. A trimodal stationary phase column, offering a combination of reversed-phase/cation-exchange/anion-exchange mechanisms, was employed to enhance selectivity and retention of the highly polar analytes. The use of ELSD enabled the detection of non-chromophoric inorganic ions with satisfactory sensitivity and reproducibility. Validation of the method was performed in accordance with ICH guidelines, demonstrating acceptable linearity (*R*^2^ > 0.99), precision (RSD < 10%), accuracy (recoveries between 95–105%) and LOD values suitable for routine quality control. The method showed adequate robustness in the studied formulations. This novel application of a trimodal column with HPLC-ELSD offers a powerful alternative to ion chromatography or ICP-MS, providing a simpler and cost-effective approach for inorganic ion analysis in complex pharmaceutical matrices.

## Abstract 2

The quantification of inorganic ions such as sodium and phosphate ions in pharmaceutical formulations is crucial for ensuring the consistency and safety of the drug product.

## Graphical abstract

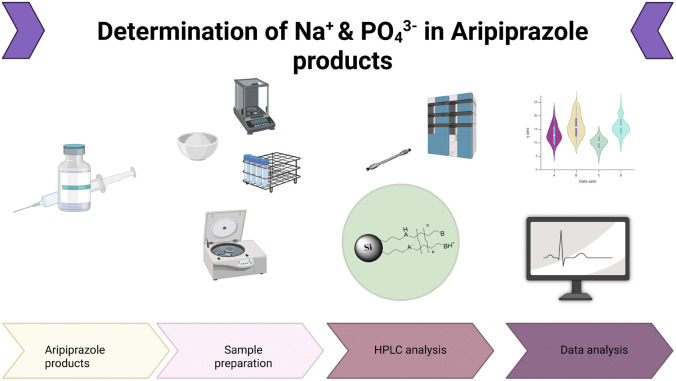

## 1. Introduction

In the pharmaceutical industry, inactive excipients play a crucial role in formulation development alongside active ingredients. They are non-active substances included in the manufacturing process or present in the final dosage form of a pharmaceutical product. Accurate identification and quantification of ionic species within excipients are essential for monitoring their content and concentration, ensuring compliance with good manufacturing practices, and detecting possible adulteration or contamination.^1^

Separation techniques and particularly high-performance liquid chromatography (HPLC) are fundamental in pharmaceutical analysis. Among them, reversed-phase HPLC (RP-HPLC) using hydrophobic stationary phases (*e.g.*, C_8_, C_18_, phenyl) with UV detection is considered the gold standard.^2^ However, RP-HPLC is less effective for polar or charged species (including inorganic anions or cations) often yielding poor retention and peak shapes. To overcome these limitations, ion-exchange and mixed-mode stationary phases are employed offering enhanced selectivity and orthogonality.^2^ Despite its usefulness, ion-exchange chromatography suffers from long run and equilibration times, limited detector compatibility, and high maintenance requirements; factors that reduce its suitability for routine pharmaceutical applications, especially when simultaneous cation and anion separation is needed.^3^

Mixed-mode stationary phases integrate multiple retention mechanisms such as reversed-phase, ion-exchange, and hydrophilic interaction chromatography (HILIC) within a single ligand on the stationary surface.^4,5^ By adjusting the mobile phase pH, the ionization of functional groups can be controlled, enabling the retention of weakly ionizable compounds. The presence of polar and ionizable groups also supports HILIC-like retention at lower acetonitrile concentrations, enhancing column capacity and analyte loading.^6,7^ Retention can be finely tuned by modifying the organic solvent content, pH, or buffer concentration. These columns offer high flexibility, orthogonality, and broad applicability for simultaneous separation of polar and non-polar pharmaceuticals, making them suitable for API, impurity, and degradation product analysis.^5,8^ They are also compatible with mass spectrometric detection.^9^

Since most pharmaceutical counterions and excipients lack UV-absorbing chromophores, detectors such as conductivity and refractive index (RI) have commonly been employed. However, these detection modes often suffer from low sensitivity or face compatibility challenges with gradient elution. ELSD and charged aerosol detection (CAD) have emerged as novel HPLC detectors.^10^ Both detection techniques are universal and suitable for non-volatile and semi-volatile compounds but the exhibits inherent non-linearity in response. Generally, ELSD provides higher limit of detection compared to CAD or conductivity detectors. The characteristics of each detection system are summarized in Table S1 (SI). The principle of operation is based on the evaporation of the column effluent using a stream of nitrogen gas introduced into a nebulizing chamber. Large droplets are removed, while the remaining aerosol is transported through a drying tube, where it is further desolvated into particles ranging from a few nanometers to several hundred nanometers in size. Detection is subsequently performed by measuring either the charged particles or the intensity of the scattered light.^10^ Coupling ion-exchange chromatography with the aforementioned detection systems presents challenges, primarily due to the low volatility of the eluents. Salt precipitation can occur, potentially damaging the nebulizer and optical cell, and leading to the need for frequent maintenance.^11^

The purpose of this research work is to develop a HPLC-ELSD method that can simultaneously separate positively and negatively charged inorganic ions (sodium and phosphate ions) in aripiprazole extended-release injectable suspensions. Compared to conventional ion-exchange chromatography, ICP-MS, and mixed-mode HILIC techniques, the proposed approach provides a balanced combination of sensitivity, robustness, and ease of implementation. It operates on standard HPLC instrumentation without requiring suppressors, high-purity argon plasma, costly equipment, or mobile phases rich in organic solvents. Although ICP-MS offers excellent elemental sensitivity, its application for sodium and phosphate determination is limited by matrix effects, space-charge interferences, high ionization potential of sodium, memory effects which can lead to unstable signals and poor quantitation.^12^ These techniques also demand rigorous sample preparation and frequent maintenance, making them less practical for routine quality control of complex pharmaceutical matrices.^13^ To the best of our knowledge, no other analytical method has been reported for this purpose up to date. The complexity of samples containing aripiprazole API and other species supports the use of trimodal-based stationary phase (HILIC, cation- and anion-exchange properties), which offers significant flexibility in adjusting various parameters to enhance analyte selectivity (Fig. S1). The developed analytical scheme was validated according to ICH guidelines and finally applied to both generic and reference products.

## 2. Experimental

### 2.1 Chemicals and materials

Sodium nitrate standard solution for IC (1000 μg per mL Na^+^, phosphate free) (TraceCert) and potassium phosphate standard solution for IC (1000 μg per mL PO_4_^3−^, sodium free) (TraceCert) were purchased from Supelco. Ammonium formate (HCOONH_4_) (>99%) and sodium dihydrogen phosphate (NaH_2_PO_4_) were obtained from Sigma-Aldrich (Laramie, WY, USA). Formic acid (≥99%) and acetonitrile (ACN) (gradient grade) were purchased from Carlo-Erba (Val-de-Reuil, France) and Honeywell (Morris Plains, NJ, USA), respectively. Excipients such as mannitol and carboxymethyl cellulose were provided by USP and Ashland (Delaware, Wilmington, USA). Purified water was obtained from a production plant (Stilmas S.p.A.). The aqueous mobile phase was filtrated though Durapore membrane filter (0.45 μm).

### 2.2 HPLC instrumentation and conditions

All separations were performed on an integrated HPLC system (all-in-one) Shimadzu Prominence-I LC-2030C 3D (Kyoto, Japan) equipped with a quaternary low-pressure gradient pump, a thermostated autosampler, a column oven, an FCV-12AH valve and an ELSD LTIII detector. The instrument control and data acquisition were performed *via* LabSolutions® software (version 5.106). All analyses were performed on Amaze TH mixed-mode (250 × 4.6 mm, 5 μm, 100 Å) (HELIX Chromatography, Illinois, USA) analytical column. During the optimization step, the mobile phase was composed of ACN and an aqueous solution HCOONH_4_, with the pH adjusted to 3.2 using formic acid. The optimal chromatographic conditions included 20 mM HCOONH_4_ in the aqueous phase (the pH adjusted to 3.2 using formic acid)/ACN, 70/30% v/v. The column temperature was maintained at 40 °C, the mobile phase flow rate was set at 1 mL min^−1^, and the injection volume was 20 μL. The autosampler temperature was maintained at 25 °C. The ELSD conditions included a drift tube temperature of 70 °C, with nitrogen (N_2_) used as the nebulizing gas at a pressure of 3.2 bar. To prevent detector contamination, the mobile phase was diverted to waste (through an FCV-12AH valve) during the first 4 minutes of the analysis.

### 2.3. Preparation of solutions for system suitability test (SST)

For the preparation of the system suitability solution, 0.5 mL of phosphate and 1 mL of sodium individual stock solutions (1000 μg mL^−1^ each) were transferred to a 10 mL volumetric flask and diluted to the volume with water resulting to a mixture of 50 and 100 μg mL^−1^ of phosphate and sodium ions, respectively.

### 2.4. Preparation of solutions for method validation

#### 2.4.1 Preparation of solutions for evaluation of specificity

As placebo a mixture containing all components of the formulation, except for those serving as the sources of sodium and phosphate was prepared. For the preparation of placebo solution, 120 mg of placebo powder was weighed and transferred to a 15 mL Falcon tube and 5 mL of water was added and sonicated for 5 min. The solution obtained was centrifuged at 20k rcf for 15 min. A portion of the supernatant was then filtered through a 0.45 μm PTFE syringe filter prior to HPLC-ELSD analysis.

Individual solutions of carboxymethyl cellulose sodium, mannitol, and sodium hydrogen phosphate were prepared by weighing the appropriate amounts of each solid, transferring them to separate volumetric flasks, and diluting with water to obtain final concentrations corresponding to the product composition.

#### 2.4.2 Preparation of solutions for evaluation of linearity

Linearity was evaluated for each analyte, and the respective solutions were prepared at various concentration levels, and each was injected in triplicate. By appropriately diluting the stock solutions of each ion in water, solutions with phosphate/sodium ion concentrations of 25/50, 37.5/75, 50/100, 62.5/125, and 75/150 μg mL^−1^, respectively, were prepared. These corresponded to a concentration range of 50–150% relative to the specification limit.

#### 2.4.3 Preparation of solutions for evaluation of precision-repeatability

A volume of 0.5 mL of the suspension was diluted 10-fold with water and then transferred into a falcon tube. The solution was shaken vigorously and then centrifuged at 20k rcf for 15 min. A portion of the supernatant was then filtered through a 0.45 μm PTFE syringe filter prior to HPLC-ELSD analysis. Six independent sample solutions were prepared.

#### 2.4.4 Preparation of solutions for evaluation of accuracy

The accuracy of the method was examined by spiking to the placebo samples with standard solutions of the analytes. An amount of 240 mg of placebo powder was weighed and transferred to a 15 mL falcon tube. Subsequently, 10 mL of aqueous solution of mixture of phosphate and sodium ions was added and the mixture was shaken vigorously and then centrifuged at 20k rcf for 15 min. A portion of the supernatants were then filtered through a 0.45 μm PTFE syringe filter prior to analysis. Three series of samples at three concentration levels of 50%, 100% and 150% with respect to the target concentration of each analyte.

### 2.5. Analysis of reconstituted suspension of aripiprazole product

A volume of 0.5 mL of the reconstituted suspension was diluted 10-fold with water and then transferred into a falcon tube. The solution was shaken vigorously and then centrifuged at 20k rcf for 15 min. A portion of the supernatant was then filtered through a 0.45 μm PTFE syringe filter prior to HPLC-ELSD analysis. Six independent sample solutions were prepared.

### 2.6 Calculations

The % content (mg mg^−1^) of the phosphate and sodium ions in the product suspension was calculated as follows:
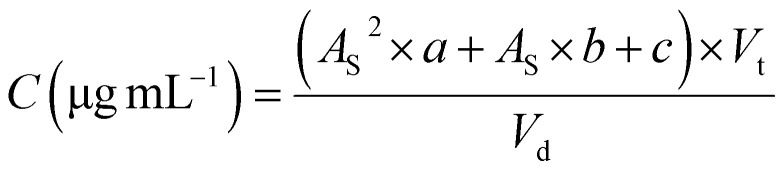
where *C*_s_ is the concentration (in μg mL^−1^) of the respective ion in sample, *A*_S_ is the peak area of phosphate or sodium ions, *V*_t_ is the volume of the sample used for dilution and *V*_d_ is the volume of the volumetric flask used for sample preparation.

## 3. Results and discussion

### 3.1 Optimization of separation conditions

Method scouting experiments were conducted using the Amaze TH mixed-mode stationary phase, which integrates HILIC characteristics with both weak cation- and anion-exchange functionalities. The primary retention mechanisms involve dipole–dipole interactions and weak electrostatic forces between the charged analytes and the reversibly charged stationary phase. Due to the intricate nature of ions and the characteristics of the mixed-mode column, it was anticipated that several factors would influence the method's retention and selectivity.

**Fig. 1:**
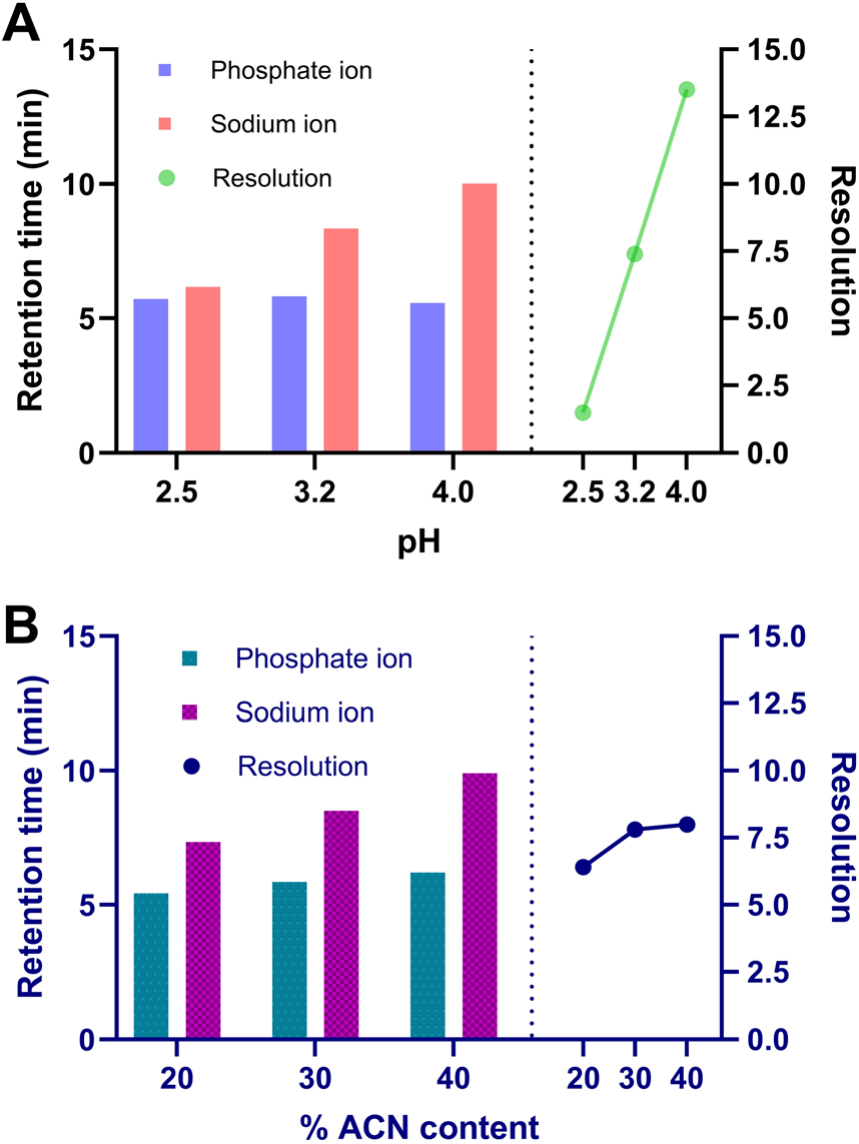
(A) Effect of the buffer pH and (B) % ACN content of the mobile phase on the retention time and resolution of phosphate and sodium ions.

According to the column manufacturer, optimal separations are typically achieved using a mobile phase composed of aqueous HCOONH_4_ buffer solution and ACN. The pH of the buffer solution was evaluated within the range of 2.5 to 4.0. Fig. 1A indicates the retention time of each ion and the resolution dependence of the buffer pH. All other experimental conditions were maintained consistently at different pH values. The retention time of phosphate ion remains almost unaffected in the tested pH range due to its persistent monovalent negative charge (p*K*_a_1__ = 1.96, p*K*_a_2__ = 7.12, and p*K*_a_3__ = 12.32) and the constant positive charge of the amino groups of stationary phase. On the other hand, longer retention times were observed for sodium ion due to the dissociation of weak anion-exchange groups (–COO^−^) of the stationary phase which resulted in stronger coulombic attraction. At lower pH value the resolution (*R*_s_) between ions was less than 1.5 but it increased linearly at higher pH values. The pH value of 3.2 was selected as optimum since it provides lower retention time of the analytes and adequate resolution (*R*_s_ = 7.4). Additionally, this value is withing the buffering range of the HCOONH_4_ solution and the recommended pH range of the analytical column.

The ACN content was investigated between 20–40% v/v. As can be observed in Fig. 1B, the phosphate ion exhibited stronger retention than the sodium ion. This is likely due to the weak anion-exchange nature of the anion-exchange domains in the Amaze TH stationary phase. The ionization of these binding sites decreases with increasing ACN content, thereby weakening the electrostatic interactions with anions. In contrast, the cation-exchange domains of the column as strong cation exchangers, with their ionization remaining stable despite changes in the organic solvent content. Similar findings have been previously reported by Liu *et al.*^7^

In ion-exchange mode, the retention of ionic species will reduce with the increase of buffer ionic strength. The capacity factor (*k*) of an analyte typically exhibits a relationship with the mobile phase concentration described by the following equation:log *k* = log *k*_o_ − *n* log *C*where *n* is a constant depending on the ion-exchange mechanism and the ion charge, *k*_o_ is the capacity factor at a reference eluent concentration and *C* is the concentration of the competing ion in mobile phase.

**Fig. 2:**
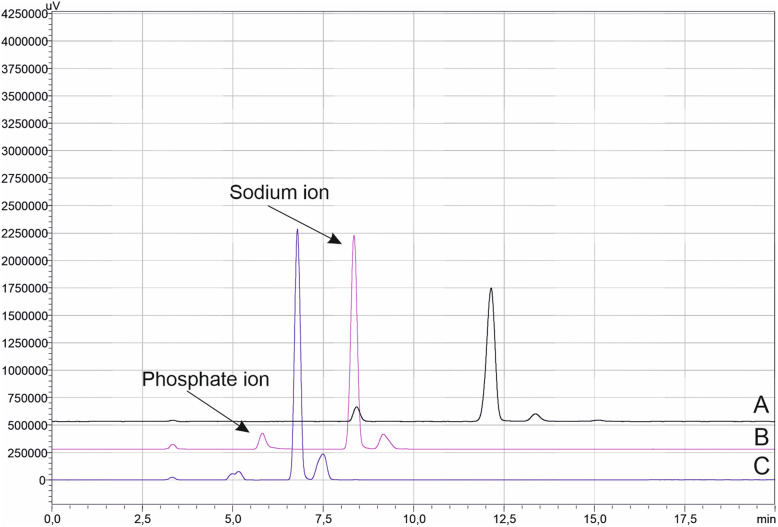
Effect of HCOONH_4_ concentration, (A) 10 mM, (B) 20 mM and (C) 30 mM on the separation of the analytes.

Fig. 2 presents chromatograms illustrating the separation of the analytes under varying concentrations of buffer solution. As the HCOONH_4_ concentration increased from 10 to 30 mM, a decrease in the retention times of the ions was observed. At higher salt levels, reduced separation efficiency and partial peak splitting of the phosphate ion were observed. Fundamentally, the retention time of an ion reflects the change in the standard Gibbs free energy of transfer of the analyte between the mobile phase and the stationary phase, as influenced by variations in the salt concentration of the mobile phase.^8^ At moderate buffer strength (20 mM, pH 3.2), sufficient ionization of both analytes and stable electrostatic interactions with the mixed-mode sites of the stationary phase were achieved, resulting in symmetrical peaks and reproducible retention. Therefore, 20 mM HCOONH_4_ at pH 3.2 was adopted for further experiments.

**Fig. 3:**
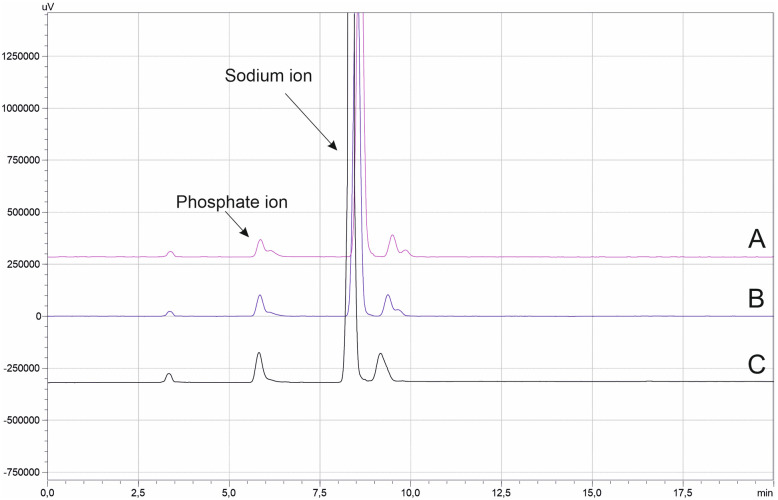
Effect of column temperature, (A) 25 °C, (B) 30 °C and (C) 40 °C on the separation of the analytes.

The effect of the column temperature on the peak symmetry of the analytes peaks was studied in the range of 25–40 °C. As is illustrated in Fig. 3, the retention time of both analytes were not changed over the examined temperature range. However, improved peak symmetry was observed for the phosphate ion at higher temperature, while the peak symmetry of the sodium ion remained almost unaffected. Thus, the value of 40 °C was selected as optimum column temperature.

### 3.2 System suitability test

The SST solution, prepared with sodium and phosphate ions, was injected into the HPLC-ELSD system under the specified chromatographic conditions to evaluate system suitability parameters. Six replicate injections were performed. The following acceptance criteria were established: (i) the % RSD of peak area for each analyte must be less than 5.0%; (ii) the % RSD of the retention time for each analyte must be below 2.0%, (iii) the resolution between phosphate and sodium ions must exceed 2.0, (iv) tailing factor should be less than 2.0 and (v) number of theoretical plates of each analyte peak must be not less than 2000. Table S2 (SI) summarizes the results of the SST, and the values obtained were found to be within the acceptable limits.

### 3.3 Method validation

The method validation encompassed the assessment of selectivity, linearity, accuracy, precision, limit of detection (LOD), limit of quantitation (LOQ) and solution stability according to ICH guidelines.^14^

The selectivity of the method was investigated by examining the chromatographic potential interferences by analysing a standard solution, sample solution, placebo and individual excipients such as carboxymethyl cellulose, mannitol, and dihydrogen phosphate ions. The analysis showed no co-elution of peaks originating from the sample components and excipients with the peaks of interest, so it can be concluded that the developed method is selective.

**Fig. 4:**
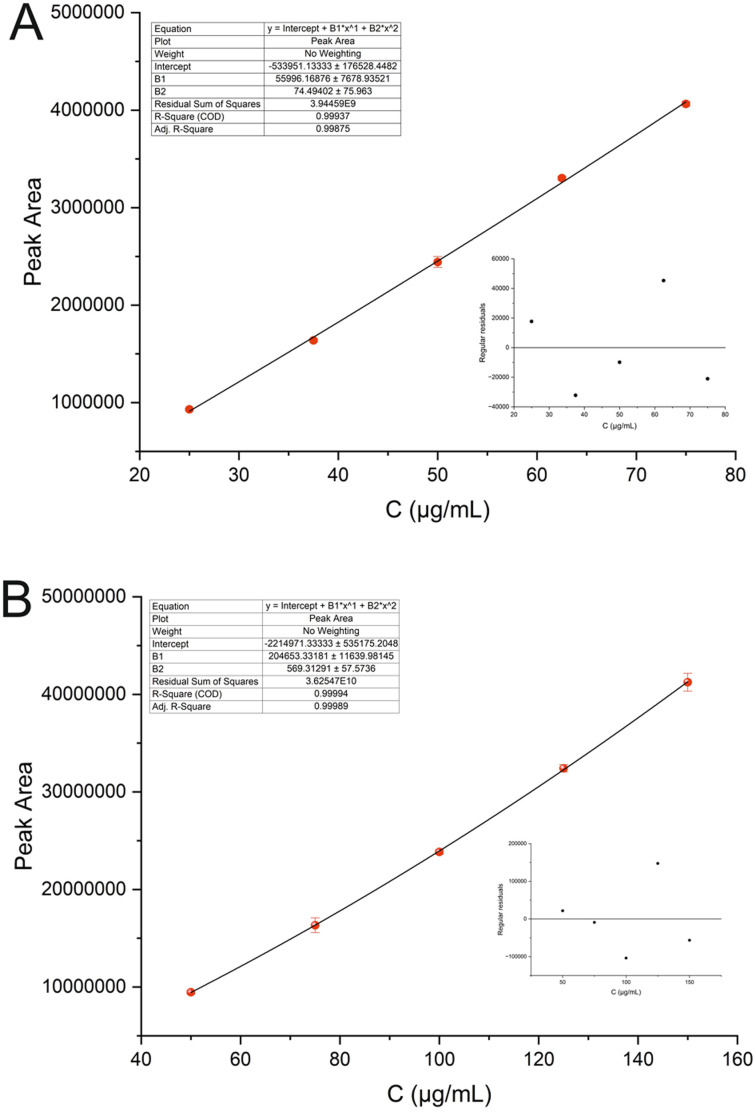
Calibration curves of (A) phosphate ion and (B) sodium ion. The inserts display the residual plots corresponding to each calibration curve.

ELSD exhibits a non-linear response, as the amount of analyte detected is related to the intensity of the scattered light through an exponential relationship.^15^ However, a linear calibration curve can be constructed after double logarithmic transformation of the exponential equation. In our case, second-order polynomial regression (area = *Ax*^2^ + *Bx* + *C*) was utilized to describe the relationship between peak area and analyte concentration as it yielded slightly higher correlation coefficient compared to logarithmic one. Calibration curves were constructed in the range of 25–75 μg mL^−1^ and 50–150 μg mL^−1^ for phosphate and sodium ion, respectively. The correlation coefficients (*R*^2^) were greater than 0.998 for both ions, indicating strong correlation within the specified concentration range (see Fig. 4). Furthermore, residuals analysis revealed no evidence of outliers or influential points across the entire concentration range, indicating homoscedasticity and supporting the reliability of the model. The normality of the residuals for the analytes was confirmed by the Shapiro–Wilk test, with *p*-values of 0.9337 and 0.9502 for phosphate and sodium ions, respectively, thereby validating the assumptions underlying the polynomial model. Other validation parameters are tabulated in Table 1. The recovery (*R*%) values for both ions ranged between 95% and 105%, with % RSD not exceeding 10%, meeting the acceptance criteria and confirming the method's accuracy.^16^ Representative HPLC-ELSD chromatograms of the analysis of samples spiked at concentration levels of 50, 100 and 150% of the specification level of each analyte are shown in Fig. S2 (SI). Intraday precision was assessed by a single analyst on the same instrument, whereas inter-day precision involved different analysts and instruments across separate days. Both repeatability and interday precision (expressed as % RSD), were below 4.1%, demonstrating the method's satisfactory precision. The limits of detection (LOD) and quantification (LOQ) were established based on signal-to-noise ratios of 3 and 10, respectively. The LODs were found to be 0.61 and 0.13 μg mL^−1^ for phosphate and sodium ion, respectively.

**Table 1:** Validation parameters of the proposed HPLC-ELSD method

Compound	Linear range (μg mL^−1^)	Calibration curve *Y* = *AX*^2^ + *BX* + *C*	*R* ^2^	Precision^a^ (% RSD)		Accuracy^b^ (% RR)		LOD (μg mL^−1^)
				Repeatability (*n* = 6)	Intermediate precision	Concentration level (μg mL^−1^)	Recovery (*R*%)	
Phosphate	25–75	*Y* = 74.49*X*^2^ + 55996*X* − 533951	0.9988	2.8^c^/3.0^d^	4.1^c^/2.8^d^	25	100.3	0.61
						50	97.5	
						75	98.3	
Sodium	50–150	*Y* = 569.3*X*^2^ + 204653*X* − 2.21 × 10^−6^	0.9999	2.2^c^/2.0^d^	4.1^c^/2.9^d^	50	97.6	0.13
						100	97.3	
						150	95.8	

a RSD: acceptance value ≤ 10%.

b Recovery: acceptance range 95–105%.

c Aripiprazole lyophilized powder.

d Aripiprazole suspension.

The stability of the SST solution (containing 50 μg mL^−1^ of phosphate and 100 μg mL^−1^ of sodium) as well as the product solutions were evaluated over a 7-day period. Multiple replicate tests were performed within 48 hours and after 7 days from the solution preparation. During this time, all solutions were stored at 25 °C and protected from light. The % difference between stability intervals for each analyte in the sample solution was calculated using the following formula:
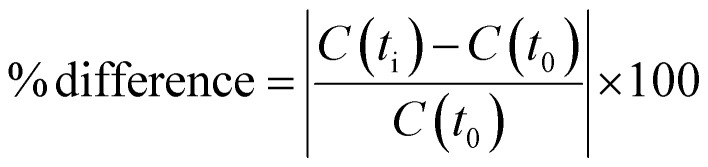
where *C*(*t*_i_) and *C*(*t*_o_) is the % content (mg mg^−1^) of each analyte in the sample solution at the respective time interval and at zero time, respectively. In all cases, the % difference was less than 3.8% which falls within the acceptance criterion of 5%, indicating good solution stability.

The experimental robustness testing is a required step of proper method validation as it is a measure of method capacity to remain unaffected by small but deliberate variations of its parameters and provides an indication of its reliability during normal usage.^17^ In our case, the robustness of the method was assessed by analyzing sample solutions. Small changes in flow rate (±0.1 mL min^−1^), column temperature (±2 °C), % buffer content in the mobile phase (±2%), buffer concentration (±10%) and pH value (±0.2) (Table S3, SI). The results showed that the % difference of the % content of both analytes in the tested sample between the modified and unmodified conditions was less than 4.8% which is within the acceptance criterion of 5%. Among the tested parameters, column temperature and buffer concentration exhibited the greatest influence while the buffer pH is sensitive for both ions. In contrast, flow rate had moderate effect, indicating good method robustness. These results confirm that the method is reliable for routine quality control.

### 3.4 Analysis of aripiprazole pharmaceutical formulations

The developed HPLC-ELSD method was applied for the determination of phosphate and sodium ions in generic and reference product (Abilify Maintena®) aripiprazole for extended release injectable suspension. The specific product is supplied as lyophilized powder that is reconstituted with the designated diluent (water for injection) immediately prior to intramuscular administration. All samples were treated according to the procedure described in Section 2.5. In the analysed samples, the % content of phosphate and sodium were varied between 491.5–535.2 and 792.3–836.1 μg mL^−1^, respectively. These values were consistent with the expected levels based on formulation composition, confirming the method's suitability for routine quality control of this product type.

## 4. Conclusions

In this study, we present a novel HPLC method employing a mixed-mode column in combination with a ELSD for the simultaneous separation and determination of phosphate and sodium ions in aripiprazole for extended-release injectable suspension. Mixed-mode column offers multiple interaction mechanisms, which provide unique opportunities but also present challenges in method development. The Amaze tri-modal column – featuring HILIC, cation- and anion-exchange properties – demonstrated distinctive selectivity in this study. To optimize the method and gain insight into the separation and retention mechanisms, we investigated the effects of organic solvent content, buffer pH and concentration, and column temperature. The validated method demonstrated excellent was validated by assessing its selectivity, linearity, accuracy, precision, robustness, and solution stability, confirming its reliability and sensitivity. Beyond this specific application, the approach offers broader utility as it provides a cost-effective alternative for ion quantification in pharmaceutical QC laboratories that may not have access to ICP-MS or ion chromatography instrumentation. Given the mixed-mode column's tunable retention mechanisms, similar approaches can be readily adapted for the analysis of other pharmaceutical formulations containing ionic excipients or counterions. This demonstrates the potential of the proposed method as a flexible and efficient platform for ion analysis across diverse drug matrices.

## Author contributions

Alexandra Bitziou: investigation, formal analysis, data curation, validation. Theodora Fotiou: conceptualization, supervision, methodology, project administration, writing – review & editing. Constantinos K. Zacharis: conceptualization, methodology, supervision, visualization, Writing – original draft, writing – review & editing.

## Conflicts of interest

There are no conflicts to declare.

## Supplementary Material

RA-015-D5RA07182H-s001

## Data Availability

The data supporting this article have been included as part of the supplementary information (SI). Supplementary information: presentation of trimodal stationary phase, chromatograms of accuracy test, system suitability values, raw data of robustness test. See DOI: https://doi.org/10.1039/d5ra07182h.

## References

[cit1] Kapoor D. U., Pareek A., Sharma M., Prajapati B. G., Suttiruengwong S., Sriamornsak P. (2025). Eur. J. Pharm. Biopharm..

[cit2] Mattrey F. T., Makarov A. A., Regalado E. L., Bernardoni F., Figus M., Hicks M. B., Zheng J., Wang L., Schafer W., Antonucci V., Hamilton S. E., Zawatzky K., Welch C. J. (2017). TrAC, Trends Anal. Chem..

[cit3] Poole C. F. , Yu L. and Sun Y., Ion-Exchange Chromatography and Related Techniques, 2024, pp. 1–23

[cit4] Wolrab D., Frühauf P., Kolderová N., Kohout M. (2021). J. Chromatogr. A.

[cit5] Lemasson E., Bertin S., Hennig P., Lesellier E., West C. (2018). J. Chromatogr. A.

[cit6] Amaze HPLC Columns, HELIX Chromatography, https://helixchrom.com/products/hplc-columns/amaze/, accessed 4 July 2025

[cit7] Liu X., Pohl C. A. (2010). J. Sep. Sci..

[cit8] Zhang K., Dai L., Chetwyn N. P. (2010). J. Chromatogr. A.

[cit9] Strege M. A., Stevenson S., Lawrence S. M. (2000). Anal. Chem..

[cit10] Magnusson L. E., Risley D. S., Koropchak J. A. (2015). J. Chromatogr. A.

[cit11] Karu N., Dicinoski G. W., Haddad P. R. (2012). TrAC, Trends Anal. Chem..

[cit12] Agatemor C., Beauchemin D. (2011). Anal. Chim. Acta.

[cit13] Sample Preparation Problem Solving for Inductively Coupled Plasma-Mass Spectrometry with Liquid Introduction Systems: Solubility, Chelation, and Memory Effects, https://www.spectroscopyonline.com/view/sample-preparation-problem-solving-inductively-coupled-plasma-mass-spectrometry-liquid-introduction?utm_source=chatgpt.com, accessed 6 October 2025 PMC4550584 26321788

[cit14] Committee for Medicinal Products for Human Use ICH Q2(R2) Guideline on validation of analytical procedures, https://www.ema.europa.eu/en/about-us/contacts-european-medicines-agency/send-question-european-medicines-agency, accessed 21 March 2024

[cit15] Megoulas N. C., Koupparis M. A. (2005). Crit. Rev. Anal. Chem..

[cit16] Method Validation in Pharmaceutical Analysis, ed. J. Ermer and P. Nethercote, Wiley, 2014

[cit17] International Conference on Harmonization (ICH) of Technical Requirements for Registration of Pharmaceuticals for Human Use, Topic Q2 (R1): Validation of Analytical Procedures: Text and Methodology, Geneva, 2005

