# Supplementary material for: Efficient HPLC-ELSD analysis of sodium and phosphate in aripiprazole extended-release injectable suspensions using trimodal column technology
Source: RSC Adv. 2025 Oct 28;15(48):41108–14. doi: 10.1039/d5ra07182h (PMC12559994; doi:10.1039/d5ra07182h)
Supplement: RA-015-D5RA07182H-s001 [file RA-015-D5RA07182H-s001.pdf]

Supplementary Information (SI) for RSC Advances.
This journal is © The Royal Society of Chemistry 2025

Electronic Supporting Information

Efficient HPLC-ELSD Analysis of Sodium and Phosphate in Aripiprazole Extended-Release Injectable Suspensions Using Trimodal Column Technology

Alexandra Bitziou[1,2], Theodora Fotiou[2,*], Constantinos K. Zacharis [1,*]

[1]*Laboratory of Pharmaceutical Analysis, Department of Pharmacy, Aristotle University of Thessaloniki, GR-54124, Greece*

[2] *Analytical Development laboratory, Innovative Formulations, Pharmathen SA, 31 Spartis Str., 14452 Metamorfosi Attica, Greece*

---

*Corresponding author
Theodora Fotiou
Analytical Development Manager
Innovative Formulations,
Pharmathen S.A. Pharmaceutical Industry, 31 Spartis Str., 14452 Metamorfosi Attica, Greece
E-mail: tfotiou@pharmathen.com

*Corresponding author
Constantinos K. Zacharis
Associate Professor
Laboratory of Pharmaceutical Analysis, Department of Pharmacy,
Aristotle University of Thessaloniki (AUTh),
GR-54124, Greece
Tel: +30 2310997663
E-mail: czacharis@pharm.auth.gr

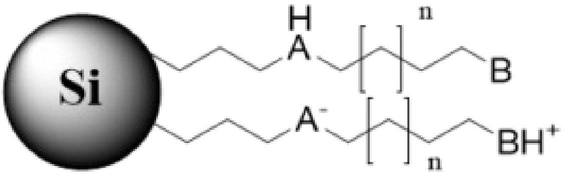

Figure S1. Schematic representation of functional groups of the trimodal stationary phase (Amaze TH mixed-mode, HELIX Chromatography). Where *n*, HA, A^-, B and BH^+ represent the carbon chain, the acid group in non-ionized state, the acid group in ionized state, basic group in non-ionized state and basic group in ionized state, respectively.

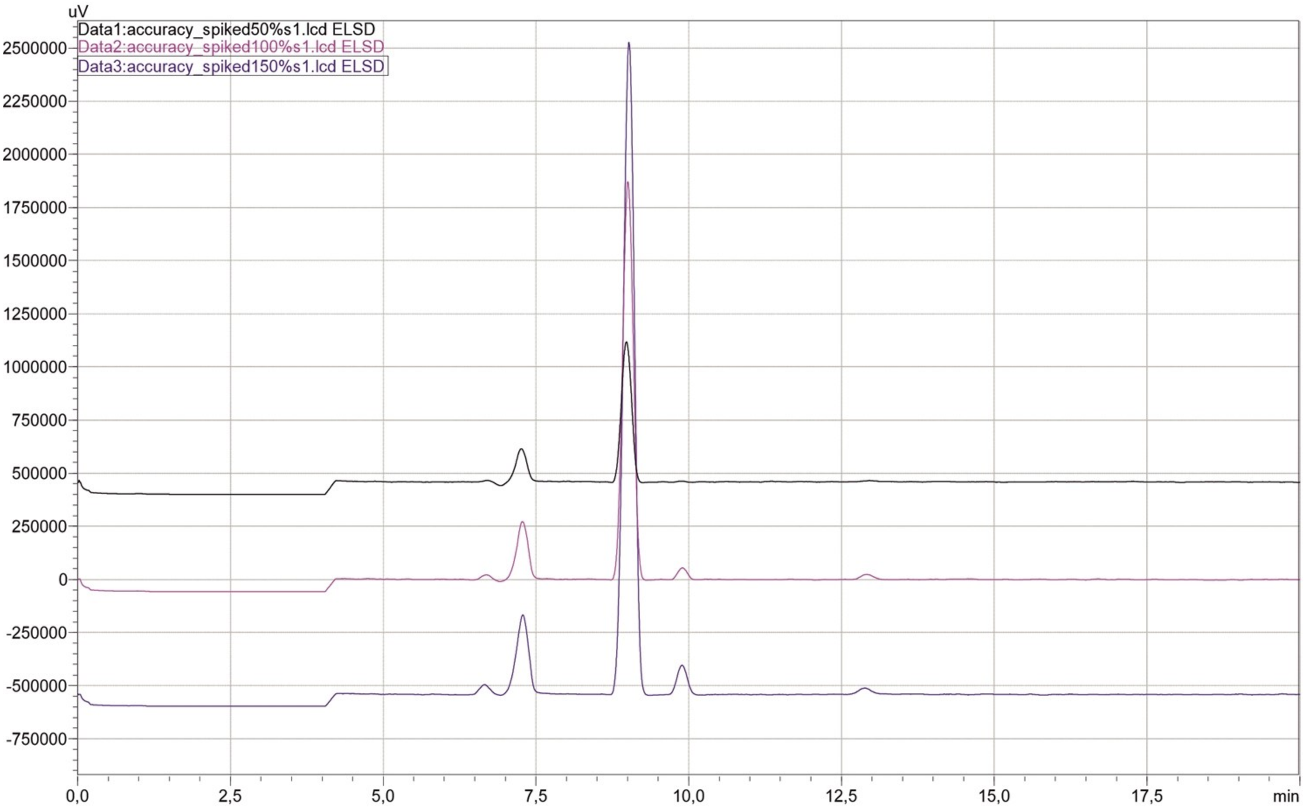

Figure S2. Representative HPLC-ELSD chromatograms of the analysis of samples spiked at concentration levels of 50, 100 and 150% of the specification level of each analyte. To prevent detector contamination, the mobile phase was diverted to waste during the first 4 minutes of the analysis.

Table S1. Comparison of common detection techniques for this type of analysis.

Detector	Detection principle	Linearity	Sensitivity (LOD)	Volatility compatibility	Advantages	Limitations
ELSD	Light scattering from evaporated solvent particles	Non-linear	Moderate	Non-volatile analytes	Universal detection; compatible with gradient elution	Non-linear response<br>higher LOD
CAD	Charged aerosol detection	Near linear	High	Non-volatile analytes	Excellent sensitivity; consistent response	Higher cost, limited volatility range
Conductivity	Measurement of ionic current	Linear	Very high	Ionic analytes only	Outstanding sensitivity for ionic species	Limited to ionic compounds
UV	Absorbance of UV light	Linear	High	UV-active analytes	Simple; cost-effective; widely available	Requires chromophore, not universal
RID	Measurement of refractive index difference between sample and reference	Linear (isocratic only)	Moderate to low	Non-volatile analytes	Universal detection; non-destructive	Not compatible with gradient elution, poor sensitivity, baseline drift

Table S2. System suitability test (SST) values.

Run	Phosphate ion		Sodium ion		R_s[a]
	**Peak area**	**R_t (min)**	**Peak area**	**R_t (min)**	
1	2503388	7.30	24529628	9.08	5.3
2	2476864	7.30	25146595	9.09	5.3
3	2526614	7.30	25064521	9.09	5.2
4	2445364	7.30	25208537	9.09	5.3
5	2498988	7.30	25701690	9.09	5.3
6	2585120	7.30	25177259	9.08	5.4
%RSD	1.9	-	1.5	-	-
T_f[b]	0.9		1.0		
N_{min}[c]	7261		11079		

[a] Resolution between Phosphate and sodium peaks
[b] tailing factor
[c] minimum number of theoretical plates

Table S3. Robustness test of the proposed HPLC-ELSD method.

	Phosphate ion		Sodium ion	
	**%Content of sample (%mg/mg)**	**% Difference with Unmodified conditions**	**%Content of sample (%mg/mg)**	**% Difference with Unmodified conditions**
**Flow rate (+0.1 mL/min)**	0.23	3.0	0.34	1.9
	0.24	3.1	0.33	3.1
**Flow rate (-0.1 mL/min)**	0.23	0.1	0.34	1.1
	0.22	3.3	0.33	2.9
**Column temperature (+2°C)**	0.21	4.6	0.34	0.0
	0.21	2.1	0.32	1.6
**Column temperature (-2°C)**	0.21	4.7	0.34	0.7
	0.21	2.2	0.33	0.4
**% Mobile phase A content (+5%)**	0.21	3.5	0.34	0.3
	0.21	2.9	0.33	0.7
**% Mobile phase A content (-5%)**	0.21	3.7	0.34	1.1
	0.21	3.7	0.32	2.3
**Mobile phase A: $HCOONH_4$ concentration (+10%)**	0.21	4.6	0.34	0.3
	0.21	3.1	0.34	1.4
**Mobile phase A: $HCOONH_4$ concentration (-10%)**	0.22	0.4	0.33	3.2
	0.22	0.6	0.32	2.8
**Mobile phase A pH (+0.2)**	0.21	4.6	0.34	0.3
	0.21	3.1	0.34	1.4
**Mobile phase A pH (-0.2)**	0.21	1.1	0.35	3.4
	0.21	2.3	0.35	4.8
